# Mitochondrial protein CMPK2 regulates IFN alpha-enhanced foam cell formation, potentially contributing to premature atherosclerosis in SLE

**DOI:** 10.1186/s13075-021-02470-6

**Published:** 2021-04-19

**Authors:** Jenn-Haung Lai, Li-Feng Hung, Chuan-Yueh Huang, De-Wei Wu, Chien-Hsiang Wu, Ling-Jun Ho

**Affiliations:** 1 Department of Rheumatology, Allergy and Immunology, Chang Gung Memorial Hospital, Lin-Kou, Tao-Yuan, Taiwan, Republic of China; 2 Graduate Institute of Clinical Research, National Defense Medical Center, Taipei, Taiwan, Republic of China; 3 Institute of Cellular and System Medicine, National Health Research Institute, Zhunan, Taiwan, Republic of China

**Keywords:** CMPK2, Atherosclerosis, Interferon alpha, Macrophages, Foam cell formation, Mitochondria

## Abstract

**Background:**

Premature atherosclerosis occurs in patients with SLE; however, the mechanisms remain unclear. Both mitochondrial machinery and proinflammatory cytokine interferon alpha (IFN-α) potentially contribute to atherogenic processes in SLE. Here, we explore the roles of the mitochondrial protein cytidine/uridine monophosphate kinase 2 (CMPK2) in IFN-α-mediated pro-atherogenic events.

**Methods:**

Foam cell measurements were performed by oil red O staining, Dil-oxLDL uptake and the BODIPY approach. The mRNA and protein levels were measured by qPCR and Western blotting, respectively. Isolation of CD4+ T cells and monocytes was performed with monoclonal antibodies conjugated with microbeads. Manipulation of protein expression was conducted by either small interference RNA (siRNA) knockdown or CRISPR/Cas9 knockout. The expression of mitochondrial reactive oxygen species (mtROS) was determined by flow cytometry and confocal microscopy.

**Results:**

IFN-α enhanced oxLDL-induced foam cell formation and Dil-oxLDL uptake by macrophages. In addition to IFN-α, several triggers of atherosclerosis, including thrombin and IFN-γ, can induce CMPK2 expression, which was elevated in CD4+ T cells and CD14+ monocytes isolated from SLE patients compared to those isolated from controls. The analysis of cellular subfractions revealed that CMPK2 was present in both mitochondrial and cytosolic fractions. IFN-α-induced CMPK2 expression was inhibited by *Janus kinase (*JAK)1/2 and tyrosine kinase 2 (Tyk2) inhibitors. Both the knockdown and knockout of CMPK2 attenuated IFN-α-mediated foam cell formation, which involved the reduction of *scavenger receptor* class A (SR-A) expression. CMPK2 also regulated IFN-α-enhanced mtROS production and inflammasome activation.

**Conclusions:**

The study suggests that CMPK2 plays contributing roles in the pro-atherogenic effects of IFN-α.

**Supplementary Information:**

The online version contains supplementary material available at 10.1186/s13075-021-02470-6.

## Key messages

- IFN-α enhances oxLDL-induced foam cell formation in human and murine macrophages.
- IFN-α and other atherogenic factors induce CMPK2 in T cells and monocytes from SLE patients.
- CMPK2 regulates IFN-α-induced expression of SR-A and production of mtROS.

## Background

Patients with systemic lupus erythematosus (SLE) are at a much higher risk of cerebral and cardiovascular thromboembolic events than the general population. In addition to well-known thrombogenic factors, such as anti-phospholipid antibodies, the disproportionately high prevalence and progression rates of atherosclerosis in SLE patients cannot be fully explained by traditional risk factors, such as smoking, hyperlipidemia, hypertension, and diabetes [1]. Therapeutics targeting the long-term complications from early-onset atherosclerosis and cardiovascular events are necessary, as these inevitable challenges are difficult for rheumatologists to overcome [2, 3].

Dysregulation of the immune system and immune effector cells, such as T lymphocytes, B lymphocytes, and macrophages, contributes to the development of aggravated atherosclerosis and cardiovascular diseases in patients with SLE [4–9]. Some evidence suggests that IFN-α, which is elevated and correlated with SLE disease activity [10–12], may preserve pro-atherogenic effects [13–15], although relevant studies are limited. The importance of IFN-α in atherosclerosis is also supported by evidence showing that the administration of the Janus kinase (JAK) inhibitor tofacitinib, which downregulates IFN signaling, ameliorated SLE-associated vascular disorders in a murine lupus model [16, 17].

Recent studies indicate the involvement of mitochondrial machinery in triggering IFN production in SLE patients [18–20]. In addition, abundant anti-whole mitochondria autoantibodies are present in the sera of SLE patients and are correlated with disease activity and anti-DNA autoantibody levels [21]. Mobarrez et al. further observed that the majority of large microparticles and small extracellular vesicles released from apoptotic or activated cells contain mitochondria, and the number of mitochondria microparticles is positively correlated with exacerbated lupus symptoms and increased levels of anti-double stranded DNA antibodies and pro-inflammatory cytokines [22]. Furthermore, mitochondrial dysregulation contributes to the development of atherosclerosis and various cardiovascular diseases [23].

To identify and examine the molecules potentially critical for IFN-α-mediated pro-atherogenic effects in SLE patients, we investigated the roles of cytidine/uridine monophosphate kinase 2 (CMPK2). Several reasons support this choice. First, our preliminary data showed that CMPK2 was highly induced in dendritic cells after dengue virus infection, suggesting its participation in the immune response to viral infection (data not shown). Second, CMPK2 may localize in mitochondria and is associated the differentiation of macrophages, a major cell population involved in atherosclerosis processes [24, 25]. Third, in addition to the limited microarray data showing CMPK2 induction in SLE patients’ blood samples [26], further characterization of the roles of this molecule has never been performed. Accordingly, this study examined the potential impact of IFN-α on foam cell formation and atherosclerosis-related effects and the roles of CMPK2 in IFN-α-mediated pro-atherogenic effects. Our results suggest that CMPK2 plays contributing roles in the pro-atherogenic effects of IFN-α and represents a novel potential therapeutic target for SLE that should be further evaluated.

## Materials and methods

### Cell culture and reagents

The THP-1 human monocytic cell line and Raw 264.7 murine macrophage cell line were obtained from the Bioresource Collection and Research Center, Taiwan. THP-1 cells were incubated with 30 ng/ml PMA (Sigma-Aldrich, St. Louis, USA) in RPMI for 3 days to induce their differentiation into THP-1-derived macrophages (TDMs). Raw 264.7 cells were cultured in Dulbecco’s modified Eagle *medium* (DMEM, HyClone) containing 10% fetal calf serum (FCS). Oxidized low-density lipoprotein (oxLDL) and 1,19-dioctadecyl-3,3,3′,3′-tetramethylindocarbocyanine perchlorate (Dil)-oxLDL were purchased from Kalen Biomedical (Germantown, MD, USA). LDL was obtained from Alfa (Thermo Fisher Scientific, Heysham, Lancashire, UK). Cholesterol crystals (CCs) were prepared according to a previous report [27]. Human and mouse IFN-α and interferon gamma (IFN-γ) was obtained from PBL Biomedical Laboratories (Piscataway, NJ, USA). Several Toll-like receptor (TLR) agonists, lipopolysaccharide (LPS), Pam3CSK4, poly(I:C), CpG ODN1826, CpGODN1585, and the Janus kinase (JAK)1/2 inhibitor ruxolitinib were purchased from Invitrogen (Hong Kong Science Park, Pak Shek Kok, Hong Kong.). BMS-986165, a tyrosine kinase 2 (TYK2) inhibitor, was purchased from MedChemExpress (Monmouth Junction, NJ, USA). Mitogen-activated protein kinase (MAPK) inhibitors, including PD98059, SP600125, and SB203580, were obtained from Calbiochem (Darmstadt, Germany), and AG490, a JAK1 inhibitor, was acquired from TOCRIS. Anti-CMPK2 and anti-TOMM20 antibodies were purchased from Abcam (Cambridge, UK). Anti-cleaved interleukin-1β (IL-1β) antibody was obtained from Cell Signaling (Beverly, MA, USA). A *scavenger receptor* class A (SR-A) Ab (anti-SR-A) was purchased from Santa Cruz (Santa Cruz, CA, USA). Unless specified, all other reagents were from Sigma Aldrich.

### Preparation of human primary cells and mouse bone marrow-derived macrophages (BMDMs)

Peripheral blood mononuclear cells (PBMCs) were prepared from buffy coat (purchased from the Blood Bank, Taipei, Taiwan), and both CD14^+^ monocytes and CD4+ T lymphocytes were then positively selected from among the PBMCs of SLE patients or controls by using a MACS cell isolation column (Miltenyi Biotech, Auburn, USA) as described in our previous report [28]. The diagnosis of SLE was based upon 1982 diagnostic criteria, and the use of human blood samples was approved by the IRB (no. 201509825A3) of Chang Gung Memorial Hospital, Linko, Taiwan. The preparation of mouse BMDMs was performed according to a published report [29]. In brief, male C57BL/6 mice (6–12 weeks) were purchased from the National Laboratory Animal Breeding and Research Center (Taipei, Taiwan). All of the animal studies were conducted in accordance with the protocol approved by the Institutional Animal Care and Use Committee of the National Health Research Institute (NHRI) (approval number: NHRI-IACUC-107159-AC1). Bone marrow was flushed from the tibias and femurs of mouse hind legs with DMEM using a needle syringe. After washing and filtering the marrow through a 40-μm nylon cell strainer, bone marrow cells were cultured in DMEM containing 20 ng/mL macrophage colony-stimulating factor (PeproTech Inc., New Jersey, USA) for 6 days with the medium refreshed every 2–3 days. The purity of the macrophages was more than 99%, as measured by F4/80 and CD11b staining (BioLegend CNS, Inc., USA).

### siRNA transfection

BMDMs were collected and resuspended at a concentration of 1 × 10^7^ cells/ml in modified eagle’s minimum essential medium (opti-MEM, Invitrogen) containing 300 nM siRNA specifically designed for these experiments (Stealth RNAi™ siRNA, Invitrogen). Electroporation was performed using a BTX electroporator (San Diego, CA) operating with one 300 V pulse every 3 ms. The cells (2 × 10^6^) were then seeded with macrophage-conditioned medium (Invitrogen, Carlsbad, CA, USA) containing 10% FBS for 24 h before subsequent treatment. TDMs and Raw 264.7 cells were transfected with 50 nM siRNA by using lipofectamine 3000 (Invitrogen) according to the manufacturer’s instructions. Four hours after the cells were transfected, the culture medium was replaced with fresh complete medium for further experiments.

### CMPK2-knockout THP-1 cells

CMPK2 sgRNA was designed and constructed into a CRISPR/Cas9 lentivector by the National RNAi Core Facility (RNA technology platform and gene manipulation core, Academia Sinica, Taiwan). CMPK2 sgRNA lentivirus was produced after confirming the sequence and knockout (KO) efficiency. THP-1 cells were transduced by lentivirus with CMPK2 sgRNA in the presence of polybrene (4 μg/ml) for 48 h. Then, puromycin (5 μg/ml) was added into the culture medium for 10 days with regular replacement of the medium to remove untransduced cells. Subsequently, a single clone in the 96-well plates of CMPK2-KO THP-1 cells was selected after two rounds of serial dilutions. Successful KO was confirmed by Western blotting and DNA sequencing.

### Overexpression of CMPK2 in THP-1 cells

The human *CMPK2-GFP* gene was a kind gift from Dr. Chang, Zee-Fen, professor in National Taiwan University. Expression of CMPK2-GFP and GFP plasmids in THP-1 cells was done by lipofectamine 3000 transfection according to the manufacture’s instruction.

### Oil red O staining and Dil-oxLDL uptake measurement

Oil red O staining and Dil-oxLDL uptake measurements were performed according to our previous report [30]. After treatment, the cells were washed with PBS and then fixed with 10% formalin. After formalin was removed, the cells were pretreated with 60% isopropyl alcohol and stained with 0.2% oil red O solution (Sigma-Aldrich) in 60% isopropyl alcohol. The cells were examined by light microscopy (× 400), and the percentages of oil red O positive cells in 5 microscopic fields for each independent experiment were counted and calculated. For measuring Dil-oxLDL uptake, the cells were incubated with Dil-OxLDL at a concentration of 10 μg/ml at 37 °C for 24 h and then analyzed by flow cytometry.

### BODIPY staining

For intracellular staining of neutral lipid proteins, the collected cells were washed twice with cold PBS before being fixed in 4% paraformaldehyde. After 24 h of incubation at 4 °C, the cells were stained with the 0.5 μM fluorescent neutral lipid dye 4,4-difluoro-1,3,5,7,8-pentamethyl-4-bora-3a,4a-diaza-s-indacene (BODIPY 493/503) (Molecular Probes, Eugene, OR, USA) and incubated for 20 min at 37 °C. After washing with PBS and detaching the cells with 0.5 mM EDTA (Invitrogen), the level of neutral lipids in the cells was quantified by flow cytometry analysis.

### Quantitative RT/PCR (qRT-PCR)

Total RNA was isolated with TRIzol reagent (Invitrogen) as described in our previous report [31]. RNA concentrations were measured using a NanoDrop spectrophotometer (Thermo Fisher Scientific, Waltham, MA, USA). Reverse transcription was performed in a 20-μl mixture containing 2 μg of total RNA, random hexamers, a mixture containing 10× reverse transcription buffer, dNTPs, magnesium chloride, dithiothreitol, and Moloney murine leukemia virus reverse transcriptase (Invitrogen). Then, 20 ng of cDNA was amplified in a total mixture volume of 20 μl consisting of 1× KAPA SYBR FAST qPCR Master Mix (KAPA Biosystems, Boston, MA, USA) and the appropriate gene-specific primers at a final concentration of 200 nM. The primers used are shown in Supplementary Table 1. The amplification reactions were performed on a LightCycler 480 (Roche). The changes in gene expression induced by the designated stimulation in the presence or absence of inhibitors or siRNA were calculated with the following formula: fold change = 2^−Δ (ΔC t)^, where ΔCt = Ct of target gene − Ct of GAPDH, and Δ(ΔCt) = ΔCt stimulated − ΔCt control.

### Western blotting

Enhanced chemiluminescence Western blotting (Amersham, GE Healthcare Life Science, Uppsala, Sweden) was performed as previously described [31]. Briefly, proteins were separated on a 10% SDS-PAGE gel and transferred to a nitrocellulose membrane. For immunoblotting, the nitrocellulose membrane was incubated with TBS-T containing 5% nonfat milk for 1 h and then incubated overnight with Abs against individual proteins. After washing with TBS-T, the membrane was incubated with secondary Ab conjugated to horseradish peroxidase for 1 h. The membrane was then incubated with the substrate and exposed to X-ray film. After scanning, the intensities of bands in the Western blots were compared using ImageJ software.

### Flow cytometry

The method for determining the expression of cell surface markers has been previously described [32]. The cells were collected and washed twice with cold PBS and then stained for 1 h with immunofluorescence-conjugated Abs against the designated cell surface markers at 4 °C. The cells were then analyzed and quantified using flow cytometry. The data were processed and analyzed with CellQuest software (BD Biosciences).

### Preparation of mitochondrial fraction and measurement of mitochondrial ROS

A Mitochondria/Cytosol Fractionation Kit from Abcam (Cambridge, UK) was used to extract mitochondrial and cytosolic fractions. In brief, 2 × 10^7^ cells were resuspended in 0.5 ml of 1X Cytosol Extraction Buffer Mix containing DTT and protease inhibitors. After incubation on ice for 10 min, cells were homogenized in an ice-cold Dounce tissue grinder (80–100 passes with the grinder). The homogenate was centrifuged at 700×*g* in a microcentrifuge for 10 min at 4 °C and the supernatant was then centrifuged at 10,000×*g* in a microcentrifuge for 30 min at 4 °C. After this, the supernatant was collected (cytosolic fraction) and the pellet (intact mitochondria) was re-suspended in 50 μl of the Mitochondrial Extraction Buffer Mix containing DTT and protease inhibitors (mitochondrial fraction). For mitochondrial *reactive oxygen species* (mtROS) measurement, cells were incubated with 5 μM MitoSOX™ red (Invitrogen) in culture medium for 30 min at 37 °C. After washing with PBS, the cells were detached by trypsin treatment and analyzed with flow cytometry [28].

### CCK-8 cell viability measurement assay

A Cell Counting Kit-8 (CCK-8) (Dojindo Laboratories, Kumamoto, Japan) was used to measure cell viability according to our previous report [28]. In brief, BMDMs were seeded in 96-well plates and cultured overnight, and then, the medium was replaced with fresh culture medium containing various inhibitors and incubated for 24 h. CCK-8 reagents were then added and incubated for 2 h at 37 °C. The OD values for each well were read at a wavelength of 450 nm on a microplate reader. The viability of the cells (%) was determined by subtracting the OD of experiments from the OD of the blank group and dividing this value by the OD of the control group.

### Immunofluorescence staining and confocal imaging

Cells were collected and washed two times with PBS before being fixed with 4% paraformaldehyde on ice for 20 min. The fixed cells were washed and resuspended homogenously in PBS with 0.05% Triton X-100 on ice for 5 min to permeabilize the cells. The cells were washed again with PBS and blocked with PBS containing 1% mouse FcR Blocking Reagent (Miltenyi Biotech, Bergisch Gladbach, Germany) for 30 min. Primary antibodies were added and incubated with the cells for 2 h at room temperature. After removing unbound antibodies by washing, secondary antibodies conjugated with an Alexa Fluor fluorescent dye were added and incubated in the dark for 1 h at room temperature. Cell nuclei were counterstained with 1 mg/ml DAPI (4′, 6-diamidino-2-phenylindole; Sigma) at a 1:5000 dilution. Finally, 2 × 10^3^ cells were seeded on slides, air dried in the dark, and mounted with mounting reagent (FluorSave™ Reagent, EMD Millipore Calbiochem, San Diego, CA, USA) for subsequent confocal microscopy analysis. Samples were examined with a Leica TCS SP5 confocal laser scanning microscope (Leica Microsystems, Wetzlar, Germany) equipped with an HCX PL APO 3×/1.4–0.6 oil objective (Leica). Image processing and colocalization analysis were performed with Leica LAS AF software [28]. In some, cells were fixed after incubation with MitoSOX™ red. Cell nuclei were stained with Hoechst 33528 and analyzed by confocal microscopy.

### Statistical analysis

All determinants were performed in triplicate. The data from pooled donors are expressed as the means ± SEM. Statistical comparisons were performed using Student’s *t* test or one-way analysis of variance (ANOVA). When ANOVA revealed significant differences between groups, Bonferroni’s post hoc test was used to determine the specific pairs of groups that significantly differed. A *P* value < 0.05 was considered to indicate statistical significance. Asterisks indicate values that are significantly different from the relevant control (*P < 0.05, **P < 0.01, ***P < 0.001, and ****P < 0.0001).

## Results

### CMPK2 expression was increased in immune effector cells from SLE patients

**Fig. 1:**
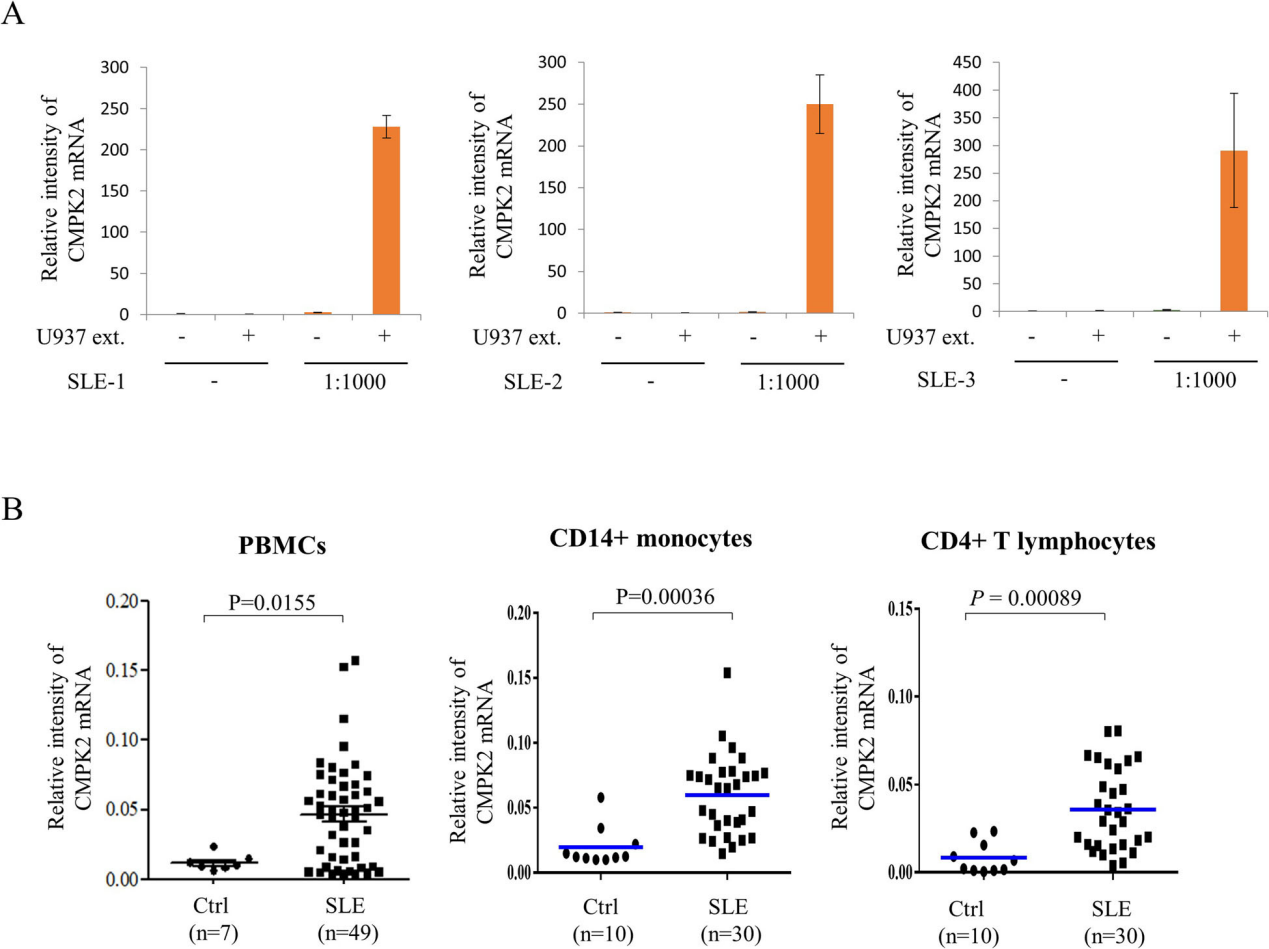
Induction of CMPK2 by immune cells from SLE patients. Peripheral blood mononuclear cells (PBMCs) were prepared from buffy coats according to the “Materials and methods” section. Cells (3 × 10^6^ cells/ml) were treated in the presence or absence of immune complexes formed by mixing U937 cellular extract (1%) and serum (1:1000 dilution) from three different SLE donors for 24 h. The cells were collected, and total RNA was prepared. Then, the levels of CMPK2 mRNA were analyzed with qPCR. The data are presented as the means ± SD (**a**). PBMCs, CD14+ monocytes, and CD4+ T lymphocytes were prepared from the whole blood of SLE patients or healthy controls. The levels of CMPK2 were determined by qPCR (**b**). CMPK2, cytidine/uridine monophosphate kinase 2

The increased formation of the immune complex and its deposition in different organs to cause immunopathology is a hallmark of SLE. PBMCs stimulated with immune complexes formed by mixing cellular extracts from U937 cells and SLE patient serum at a 1:1000 dilution, a condition modified from the method suggested by Dr. Elkon’s group [19, 33], increased the expression of CMPK2 mRNA (Fig. 1a). In accordance, the levels of CMPK2 mRNA were elevated in the PBMCs, CD4+ T lymphocytes and CD14+ monocytes from SLE patients compared to the levels in the cells from the healthy controls (Fig. 1b).

### IFN-α enhanced foam cell formation in oxLDL-stimulated macrophages

**Fig. 2:**
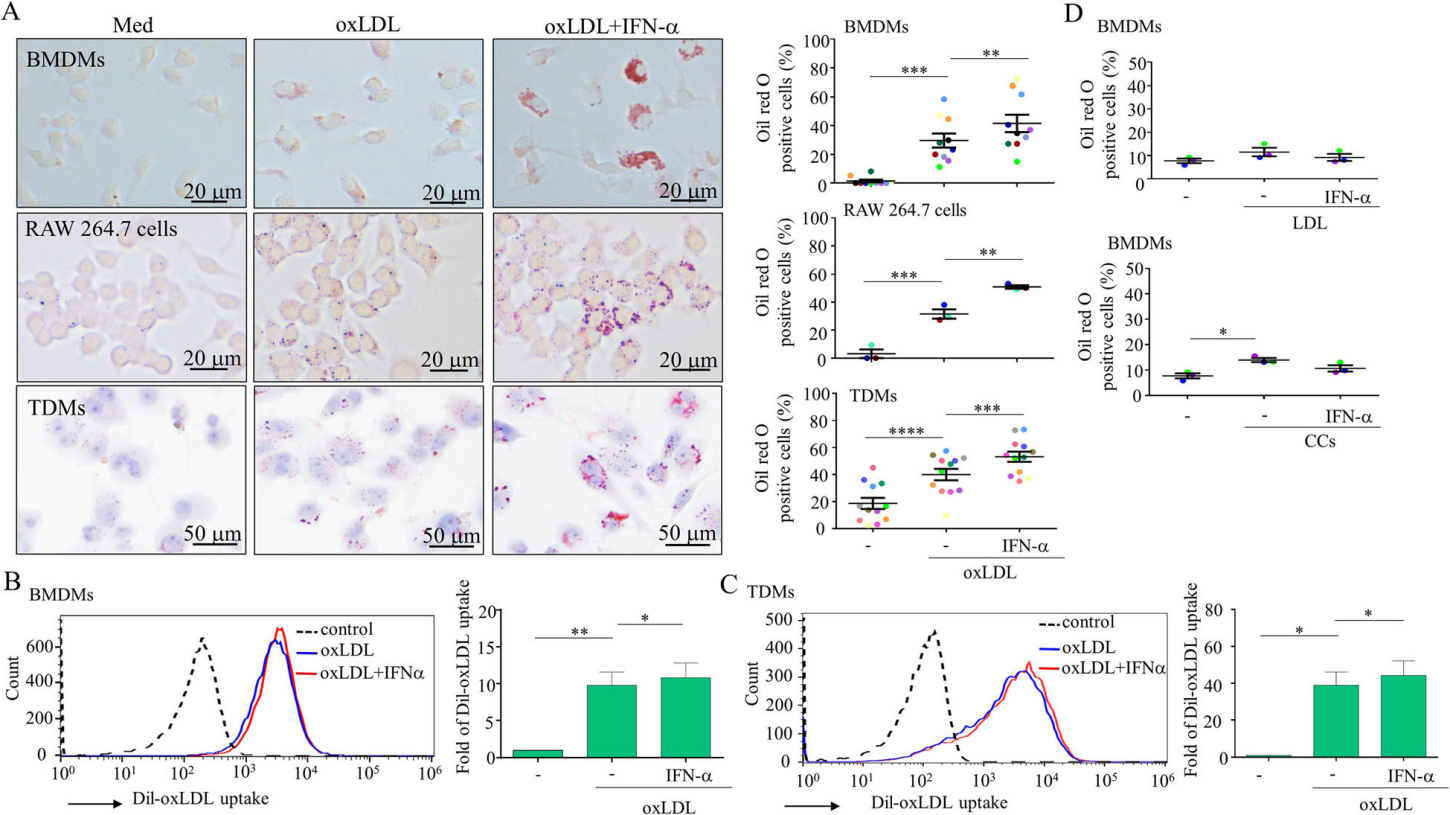
IFN-α enhanced foam cell formation. THP-1-derived macrophages (TDMs), Raw 264.7 cells, and mouse bone marrow-derived macrophages (BMDMs) were stimulated with oxLDL in the presence or absence of IFN-α, and foam cell formation was measured by oil red O staining (**a**, left). The cells were examined by light microscopy and the percentages of oil red O positive cells in 5 microscopic fields for each independent experiment were counted and calculated (**a**, right). One dot stands for an independent experiment. The extent of Dil-oxLDL uptake by BMDMs was determined and statistically analyzed and the results are shown (**b**, *n* = 6). Similar to the finding shown in **b**, the extent of Dil-oxLDL uptake by TDMs was determined (**c**, *n* = 4). Foam cell formation induced by different stimuli, including LDL and cholesterol crystals (CCs), was measured (**d**). Asterisks indicate values that are significantly different from the relevant control (*P < 0.05, **P < 0.01, ***P < 0.001 and ****P < 0.0001). n, sample numbers of independent experiments

An early study suggested that priming with IFN-α enhanced foam cell formation [34]. Following this initial report, no relevant studies examined the effect or mechanisms to determine how IFN-α may affect foam cell formation. The effects of IFN-α on foam cell formation were therefore further expanded and examined in three targeted cells: BMDMs, Raw 264.7 cells, and TDMs. As shown in Fig. 2a, IFN-α enhanced oxLDL-induced foam cell formation in human and murine macrophages. The foam cell-enhancing activity of IFN-α was relatively comparable with that of LPS and thrombin (Supplementary Figure 1). The results were correlated with the examination of Dil-oxLDL uptake, which is a measurement of scavenger receptor-mediated endocytosis, and the subsequent flow cytometry analysis of the BMDMs (Fig. 2b) and TDMs (Fig. 2c). Surprisingly, IFN-α-enhanced foam cell formation only when it was used in combination with oxLDL but not with LDL or cholesterol microcrystals (Fig. 2d).

### Regulation of CMPK2 by different pro-atherogenic factors

**Fig. 3:**
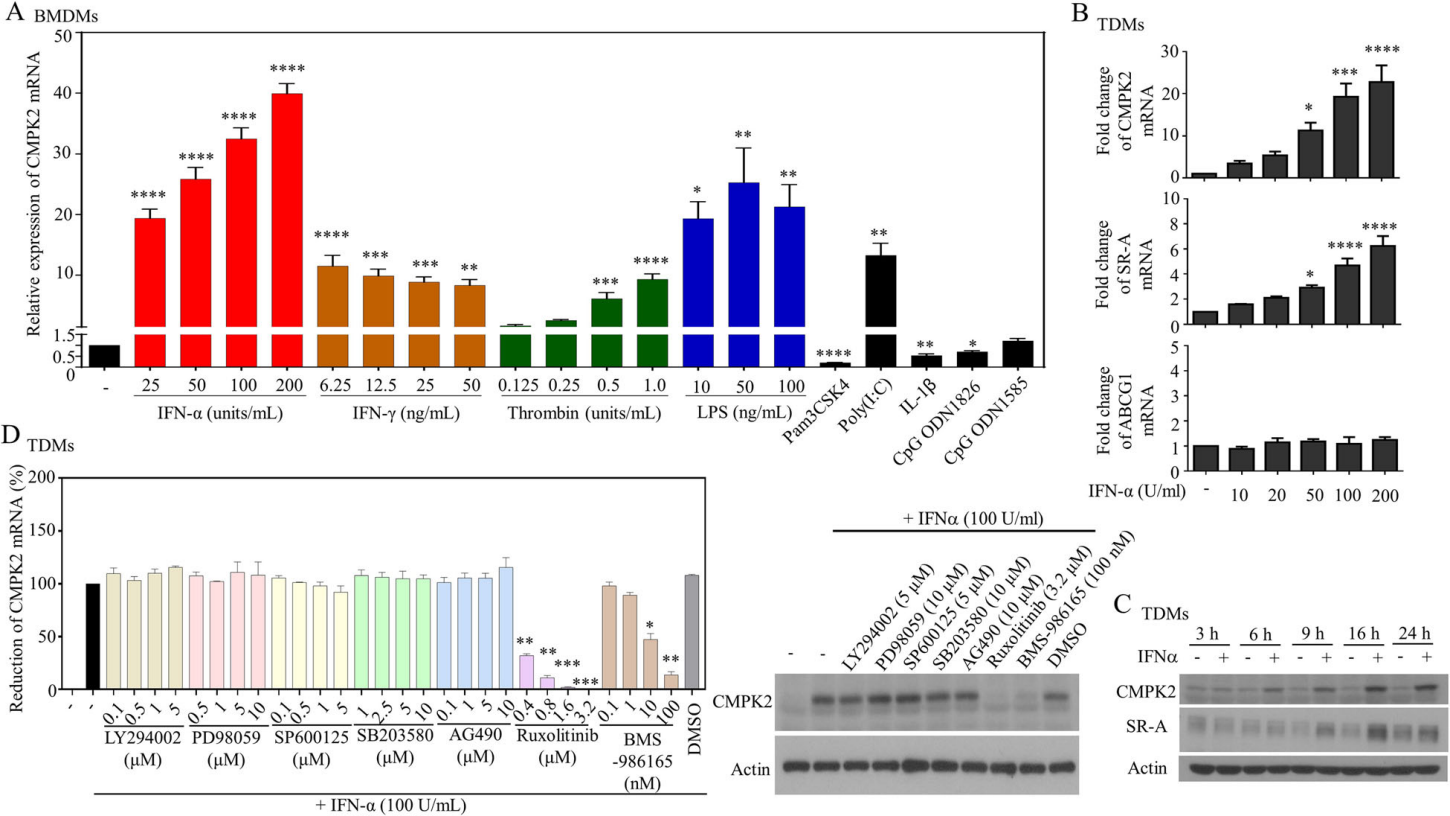
Signaling pathways involved in the activation and regulation of CMPK2. BMDMs were treated with different doses of IFN-α, IFN-γ, thrombin, lipopolysaccharide (LPS), Pam3CSK4, Poly(I:C), IL-1β, CpG ODN1826, or CpG ODN1585, and the expression of CMPK2 mRNA was measured by qPCR (**a**). The mRNA expression of CMPK2, SR-A, and ABCG1 was determined in TDMs treated with different doses of IFN-α (**b**). TDMs treated with 100 U/ml IFN-α were collected at different time points, and the protein levels of CMPK2, SR-A, and actin were measured by Western blotting (**c**). TDMs pretreated for 2 h with different doses of chemical compounds, including LY294002, PD98059, SP600125, SB203580, AG490, ruxolitinib, BMS-986165, or DMSO, were also treated with IFN-α for 24 h, and then, the cells were collected for the determination of CMPK2 mRNA by qPCR (D, left part) and in part by Western blotting (**d**, right part), respectively. Asterisks indicate values that are significantly different from the relevant control (*P < 0.05, **P < 0.01, ***P < 0.001, and ****P < 0.0001)

The effects of several pro-atherogenic stimuli on CMPK2 mRNA expression were examined. The results showed that, in addition to IFN-α, several pro-atherogenic factors, including IFN-γ, LPS, thrombin, TLR, and poly(I:C), were capable of activating CMPK2 (Fig. 3a). In contrast, treatment with *Pam3CSK4*, a TLR2/TLR1 ligand; CpG ODN1826, a TLR9 agonist; or interleukin-1β (IL-1β) at the examined doses did not induce CMPK2 mRNA in the BMDMs (Fig. 3a). These results suggest a certain specificity for CMPK2 involved in various pro-atherogenic factor-mediated effects. The less potent CMPK2-inducing activity by LPS, compared to IFN-α, might be due to kinetics and doses chosen for the study (Supplementary Figure 2). Through side-by-side comparisons of the mRNA levels of CMPK2 and the receptors involved in foam cell formation, we found that IFN-α induced the expression of SR-A mRNAs in a dose-dependent manner but did not induce ABCG1 mRNA expression (Fig. 3b). Western blotting confirmed the induction of the CMPK2 and SR-A proteins by IFN-α treatment (Fig. 3c). By confocal microscopic examination, the results suggest that CMPK2 might exist in both mitochondrial (co-localization with MitoTracker deep red) and cytosolic components of cells (Supplementary Figure 3 and further explained in Supplementary Figure 5). The examination of potential upstream molecules regulating IFN-α-induced CMPK2 activation revealed that treatment with ruxolitinib, a selective inhibitor of JAK1 and JAK2, and BMS-986165, a TYK2 inhibitor, effectively inhibited IFN-α-induced CMPK2 mRNA (Fig. 3d). In contrast, compounds targeting different signaling molecules, including those against phosphoinositide *3-kinase*, mitogen-activated protein kinases and JAK2-only, had no effect on IFN-α-induced CMPK2 expression. There were no effects on cell viability or apoptosis by these compounds at the examined concentrations, as assessed by Cell Counting Kit-8 (Supplementary Figure 4).

### Knockdown of CMPK2 attenuated IFN-α-enhanced foam cell formation

**Fig. 4:**
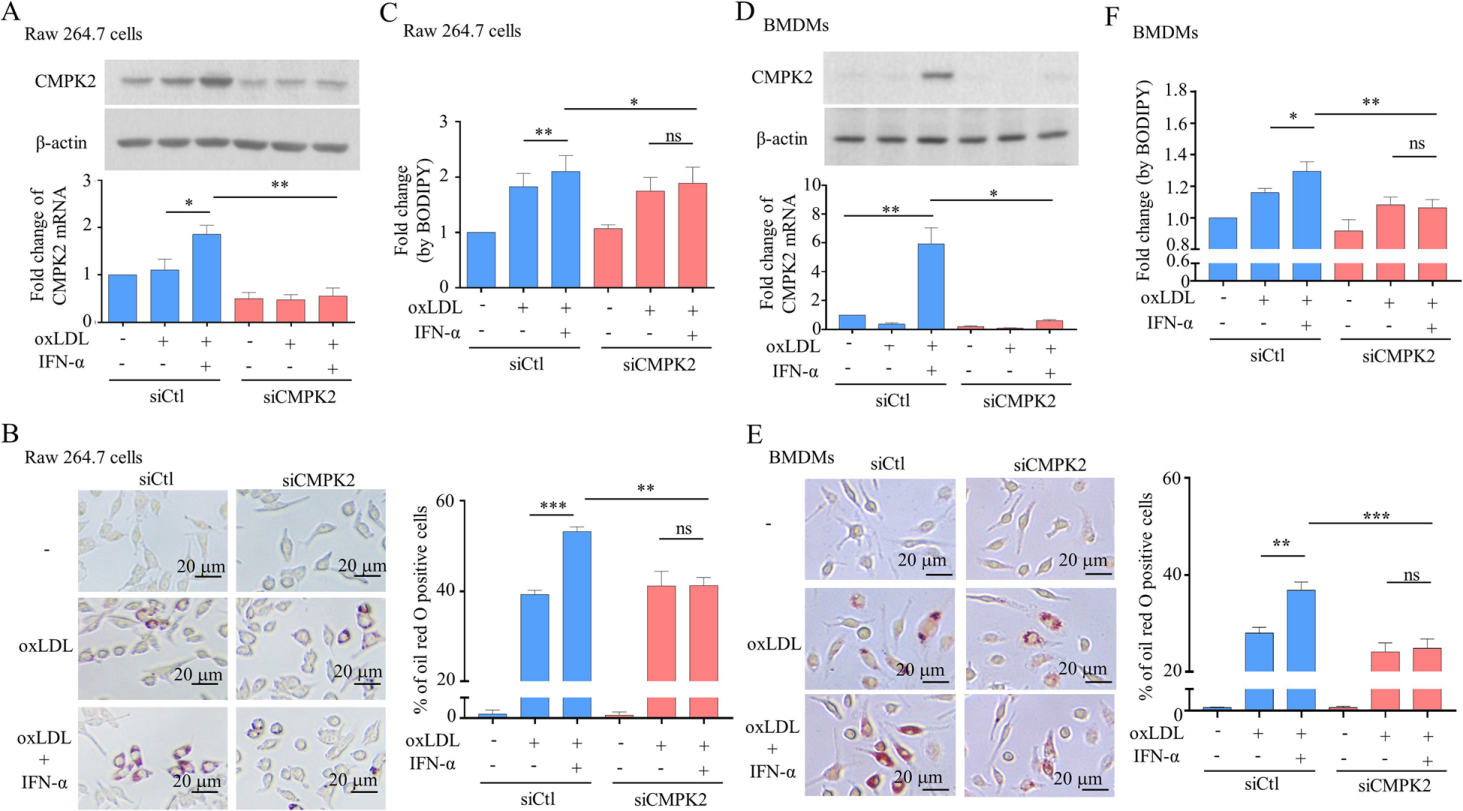
Effects of CMPK2 knockdown on BMDMs and Raw 264.7 cells stimulated by IFN-α. Raw 264.7 cells were treated with oxLDL in the presence or absence of IFN-α for 24 h. The expression of CMPK2 mRNA and protein was measured by qPCR and Western blotting, respectively (**a**). Foam cell formation was measured by oil red O staining (**b**), and BODIPY analysis was also carried out according to the description in the “Materials and methods” section (**c**). Similar analyses were performed with BMDMs (**d**–**f**). Asterisks indicate values that are significantly different from the relevant control (*P < 0.05, **P < 0.01, and ***P < 0.001)

CMPK2 siRNA was introduced into Raw 264.7 murine macrophages, and the effects of IFN-α-induced CMPK2 mRNA and protein expression were determined by qPCR and Western blot, respectively (Fig. 4a). Foam cell formation was determined by oil red O staining (Fig. 4b), and BODIPY fluorescent dye staining was analyzed by flow cytometry (Fig. 4c). The results showed that by reducing CMPK2 levels, IFN-α-enhanced foam cell formation was inhibited (Fig. 4a–c). A similar conclusion was reached when the primary BMDMs were examined (Fig. 4d–f). In support of this hypothesis, a similar conclusion was reached when similar experiments were carried out with TDMs (data not shown).

### Effects of CMPK2 knockout on IFN-α-enhanced foam cell formation

**Fig. 5:**
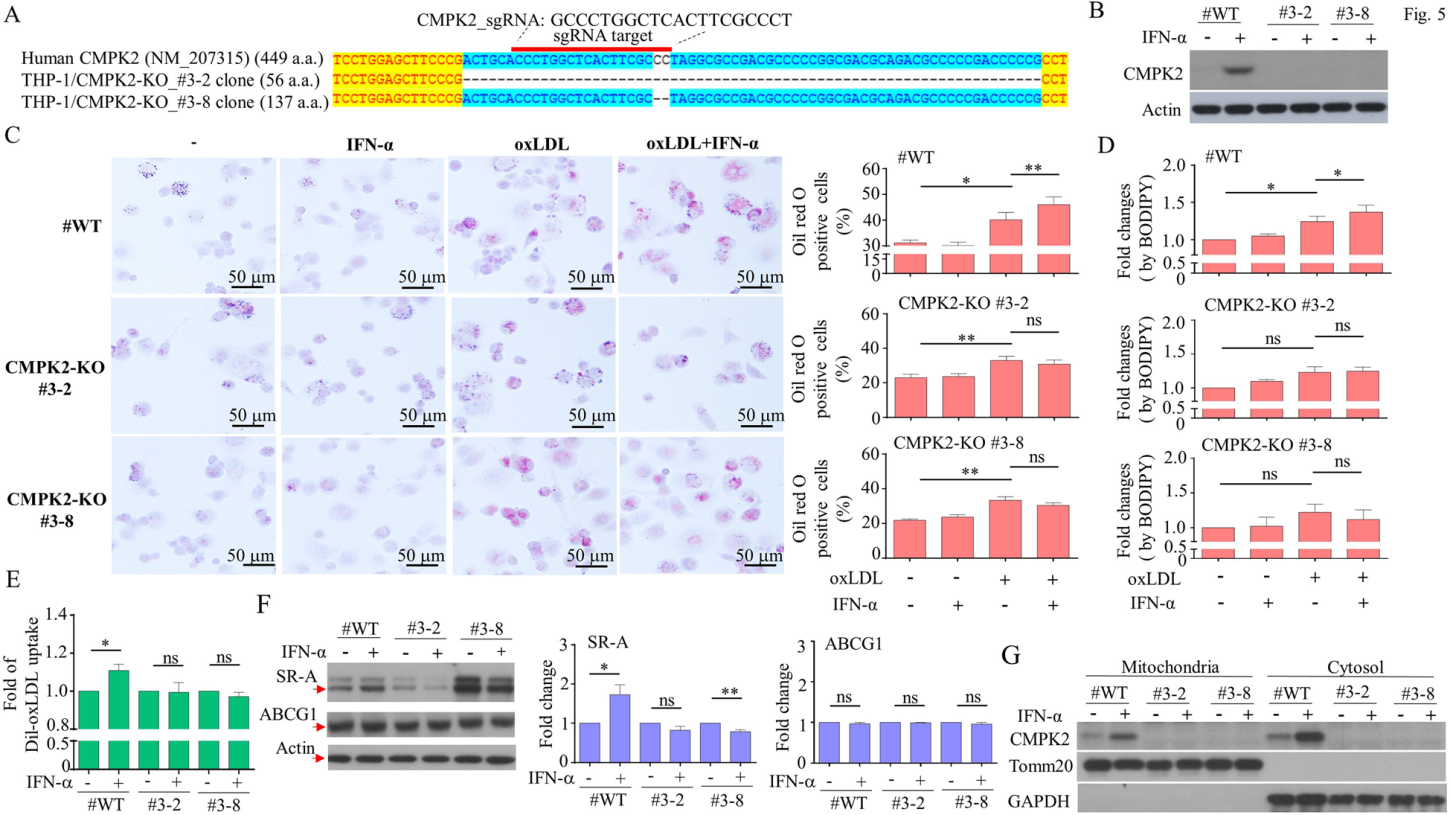
Effect of CMPK2 knockout on TDMs. The CMPK2 gene was knocked out through the CRISPR/Cas9 approach in THP-1 cells, as explained in detail in the “Materials and methods” section, and two clones, #3-2 and #3-8, were obtained; their partial sequences are shown (**a**). The expression of CMPK2 induced by IFN-α was completely abolished in CMPK2-knockout (KO) cells, as shown by Western blotting (**b**). The level of IFN-α-mediated enhancement as measured in the oxLDL-treated TDMs was significantly lower in the CMPK2-KO cells, compared to the wild-type controls (**c**). Analysis with BODIPY revealed results consistent with the results from oil red O staining (**d**). The uptake of Dil-oxLDL induced by IFN-α treatment was reduced in the CMPK2-KO cells (**e**). IFN-α-induced expression of SR-A was determined by Western blotting (**f**). IFN-α-induced expression of CMPK2 in mitochondrial and cytosolic fractions of cellular lysates was determined by Western blotting (**g**, the results from 3 independent experiments were shown in Supplementary Figure 5). Asterisks indicate values that are significantly different from the relevant control (*P < 0.05)

Because the use of siRNA to knock down specific molecules sometimes draws criticism suggesting that it inadequately suppresses the targeted protein, we used the CRISPR/Cas9 approach to knock out CMPK2 in THP-1 cells. Two CMPK2-knockout (KO) clones, #3-2 and #3-8, containing an early terminating CMPK2 gene (Fig. 5a) were generated. Treatment with IFN-α failed to induce CMPK2 in both the #3-2 and #3-8 clones (Fig. 5b). CMPK2-KO abolished the effects of IFN-α-enhanced foam cell formation (Fig. 5c). This conclusion was supported by BODIPY dye staining followed by flow cytometry analysis (Fig. 5d). The deficiency of CMPK2 also resulted in the reduction of Dil-oxLDL uptake by macrophages (Fig. 5e). The mechanism can be explained, in part, by the suppression of IFN-α-induced SR-A expression in the CMPK2-KO cells (Fig. 5f). We were surprised to find that the basal level of SR-A in clone #3-8 was higher than that in the parental clone, with no clear explanation apparent. Furthermore, the examination of cellular subfractions revealed that CMPK2 co-existed in both mitochondrial and cytosolic fractions, consistent with the observations by Zhong et al. [35], and confirmed the total elimination of CMPK2 in both fractions in CMPK2-KO clones (Fig. 5g and Supplementary Figure 5).

### CMPK2 regulated the IFN-α-induced production of mitochondrial reactive oxygen species

**Fig. 6:**
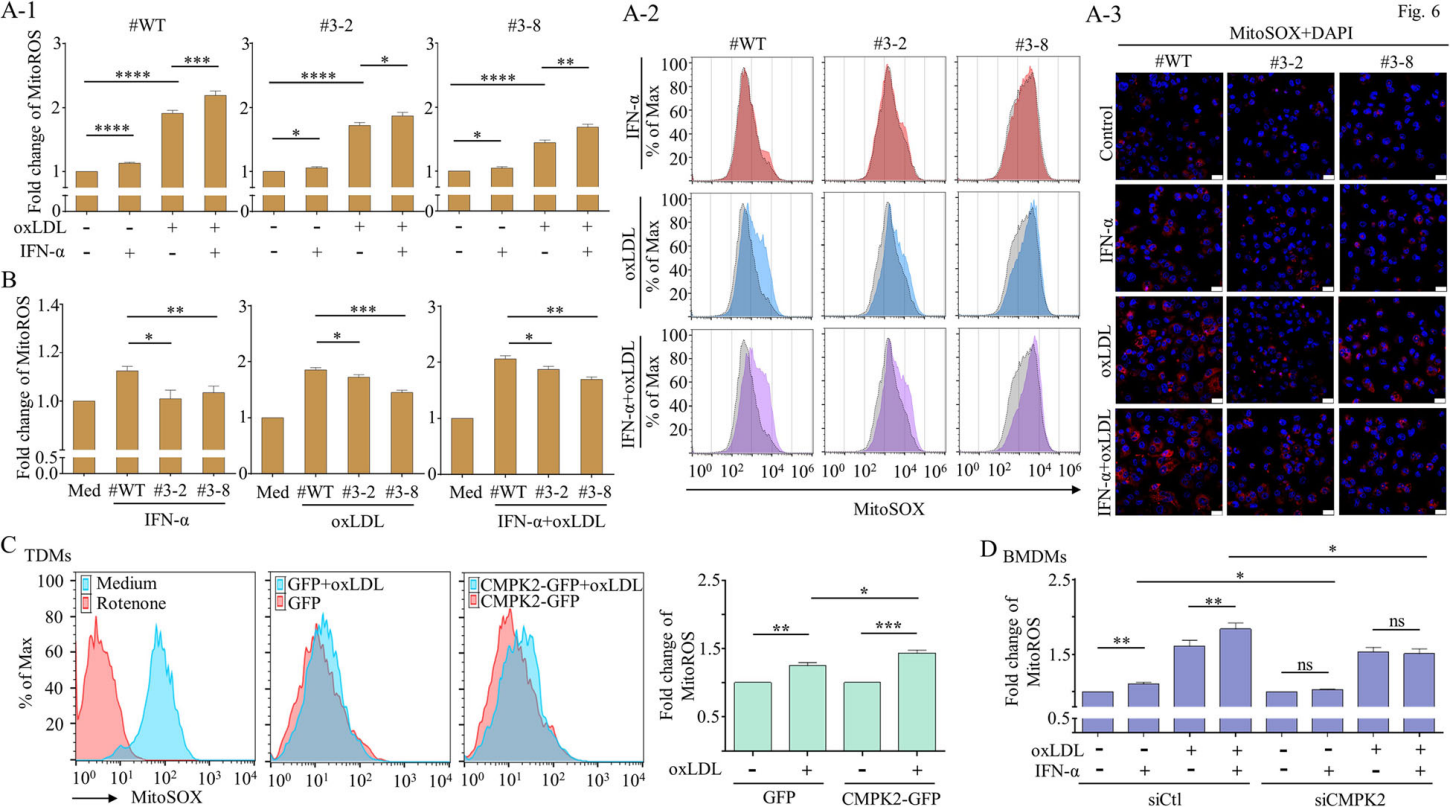
Effects of CMPK2-KO on mtROS production. Wild-type TDMs (#WT) and CMPK2-KO clones #3-2 and #3-8 were treated with IFN-α, oxLDL, or IFN-α+oxLDL for 24 h, and the generation of mtROS was detected by MitoSOX staining and then analyzed by flow cytometry (**A-1**). Representative flow cytometry results are shown in **A-2**. The unstimulated control is stained gray, and orange, blue and purple represent the conditions in the presence of different stimuli. In parallel, representative images of wild-type TDMs, clone #3-2 and clone #3-8 labeled with MitoSOX+DAPI (merged pictures) under different conditions are shown in **A-3**. The results (**A**) of the wild-type TDMs and clone #3-2 or clone #3-8 were further analyzed and compared (**B**). TDMs were transfected with GFP or CMPK2-GFP to induce the overexpression of CMPK2 and then stimulated or not with oxLDL, and the production of mtROS was measured by flow cytometry (**C**). The stimulation with rotenone served as a positive control. The results of the effects of CMPK2 knockdown on oxLDL-, IFN-α-, or oxLDL+IFN-α-induced mtROS production in BMDMs were determined (**D**). Scale bar = 25 μm in A-3. Asterisks indicate values that are significantly different from the relevant control (*P < 0.05, **P < 0.01, and ***P < 0.001)

The MitoSOX-based flow cytometry assay has been extensively used to measure mtROS formation, and this assay was undertaken for our purposes [36]. As shown in Fig. 6A-1, treatment with IFN-α or oxLDL alone increased mtROS formation compared to its effect in untreated cells, and the addition of IFN-α significantly enhanced oxLDL-induced mtROS. The deficiency of CMPK2 did not appear to have drastic statistically relevant effects on either IFN-α-, oxLDL- or IFN-α+oxLDL-induced mtROS. The representative results from flow cytometry showing MitoSOX staining and merged pictures taken by confocal microscopy showing staining with MitoSOX and DAPI are shown in Fig. 6A-2 and Fig. 6A-3, respectively. The full pictures of confocal microscopy are presented in Supplementary Figure 6. To our surprise, when the induction intensities of CMPK2 in wild-type, CMPK2-KO clone #3-2 and clone #3-8 were measured after individual treatments and compared the unstimulated control, a significant difference was identified in the fold induction of mtROS between the wild-type control and clone #3-2 and the wild-type control and clone #3-8 (Fig. 6B). The results also showed that CMPK2-KO did not significantly affect mitochondrial mass (Supplementary Figure 7). Furthermore, we found that overexpression of CMPK2 mildly but significantly increased oxLDL-induced mtROS production in the TDMs (Fig. 6C). When CMPK2 siRNA was introduced in BMDMs, the IFN-α-induced mtROS was abolished (Fig. 6D). The results suggest that CMPK2 plays, at least, a contributing role in IFN-α-, oxLDL- and IFN-α+oxLDL-induced mtROS increases. Additional experiments also suggest that CMPK2 might regulate IFN-α-stimulated inflammasome activation (Supplementary Figure 8), another mechanism associated with atherosclerosis [37].

## Discussion

Our studies demonstrated that IFN-α enhanced foam cell formation can be detected in the presence of low-dose oxLDL (20 ng/ml, in contrast to some studies with 100 ng/ml), a condition closer to physiological conditions. In addition, the IFN-α-mediated effect can be generally detected in murine primary macrophages and human and murine immortalized macrophage cell lines. Furthermore, the results suggest that such an IFN-α-mediated effect is observed only when the background stimulant is oxLDL, but not LDL or cholesterol microcrystals, indicating specificity. In addition to IFN-α, individual treatment with several other atherogenic factors induced CMPK2 expression, suggesting that CMPK2 may play roles in non-IFN-α induced mediation of atherosclerosis. Because all these CMPK2 inducers can regulate foam cell formation and atherosclerosis through different mechanisms, the data may also suggest that, when the immune response is activated in SLE patients, many factors may be potential contributors to the induction of CMPK2, that is, IFN-α might not be the only contributor critical for the CMPK2 induction observed in CD4+ T cells and CD14+ monocytes from SLE patients (Fig. 1). Nevertheless, experiments using siRNA or CRISPR/Cas9 approaches to knockdown or knockout CMPK2, respectively, confirmed the roles of CMPK2 in IFN-α-mediated foam cell formation and atherosclerosis-associated events. Although oxLDL uptake via SR-A induces the transformation of macrophages into foam cells, which is a key precursor to atherosclerosis [38–41], we found that CMPK2 is critical for IFN-α-enhanced foam cell formation because, at a minimum, it regulates SR-A expression.

The mechanisms accounting for the linkage between atherosclerosis and mitochondrial machinery remain largely unclear. The increase or accumulation of mtROS is one of the critical factors associated with atherosclerosis progression in animals and humans [42]. As expected, because oxLDL is a potent inducer of mtROS production and inflammation resulting in the progression of atherosclerosis [43], the additional effects of IFN-α in enhancing the observed effects were not very dramatic, although they were statistically significant. We observed that CMPK2-KO did not completely abolish IFN-α-mediated mtROS formation; however, in the absence of CMPK2, the intensity of IFN-α-induced mtROS production significantly decreased compared to that in wild-type cells. In support of this hypothesis, the dominant and decisive roles of CMPK2 in IFN-α-induced mtROS were evident when siRNA approaches were used to reduce CMPK2 levels in BMDMs. Although the efficacy of gene transfection in TDMs was limited, overexpression of CMPK2 significantly enhanced oxLDL-induced mtROS. To our surprise, in addition to regulating IFN-α-induced mtROS production, CMPK2 also plays roles in oxLDL-induced mtROS production. These results clearly indicate that CMPK2 is critical, at least in part, for IFN-α-induced and IFN-α-enhanced mtROS increases. In addition to mtROS production, the activation of the inflammasome pathway is important in atherosclerosis [37], and the CANTOS trial targeting the IL-1β pathway with canakinumab revealed a significantly lower rate of recurrent cardiovascular events not associated with lowering lipid levels [44–46]. With respect to this trial outcome, we observed that CMPK2-KO downregulated the IFN-α-stimulated inflammasome pathway, results reflecting part of the observations by Zhong et al. [35]. Although CMPK2 may co-existed in both mitochondrial and cytosolic fractions, its localization in mitochondria highly contributed to its immunoregulatory effects in mitochondrial machinery. Collectively, our experiments suggest that decreases in SR-A expression and mtROS production, as well as inflammasome pathway inhibition, might be critical effects of CMPK2 in regulating IFN-α-mediated pro-atherosclerosis.

There are limitations in this study. In order to solidly establish the association among IFN-α, CMPK2, foam cell formation, and atherosclerosis, the ideal experimental results will be to show that SLE patients with high IFN signature were harboring increased number of foam cells in atherosclerotic plaques and those when analyzed had high lipid index and CMPK2 levels as compared to other vascular infiltrating macrophages with lower lipid index and CMPK2 levels in healthy or SLE patients with low IFN signature. To confirm this hypothesis, reasonable numbers of vascular tissues from SLE patients with various atherosclerotic severities are required. Nevertheless, given these messages, the results show only the correlation but does not confirm that CMPK2 is actually responsible for IFN-α-enhanced foam cell formation. The correlation meets a challenge given that many non-IFN-α stimuli could induce CMPK2 expression (Fig. 3A) and many IFN-downstream and yet non-CMPK2 molecules may contribute to foam cell formation. Accordingly, the experimental approaches to knockdown or knockout CMPK2 and examine the effects in vitro may provide reasonable information explaining the roles of CMPK2 in IFN-α-enhanced foam cell formation. It is anticipated that the results from more solid approaches examining CMPK2-KO animals will provide supportive and convincing conclusions about the potential role of CMPK2 in exaggerated atherosclerosis in patients with SLE.

## Conclusions

We demonstrated that IFN-α is a potent inducer of CMPK2 and, through inducing SR-A expression, enhances oxLDL-induced foam cell formation and Dil-oxLDL uptake by macrophages. In addition to IFN-α, several triggers of atherosclerosis, including thrombin and IFN-γ, can induce CMPK2 expression, which is elevated in CD4+ T cells and CD14+ monocytes isolated from SLE patients compared to those isolated from controls. Inhibition of CMPK2 attenuates IFN-α-induced SR-A expression and IFN-α-mediated foam cell formation and the underlying mechanisms involve the regulation of IFN-α-enhanced mtROS production and inflammasome activation by CMPK2. The study suggests that CMPK2 plays contributing roles in the pro-atherogenic effects of IFN-α and CMPK2 may serve as a reasonable therapeutic target against atherosclerosis-related disorders.

## Supplementary Information

**Additional file 1: Supplementary Figure 1.** Foam cell formation induced by different stimuli. **Supplementary Figure 2.** Induction of CMPK2 by different stimuli at 6 and 24 h time points. **Supplementary Figure 3.** Determination of CMPK2 localization with confocal microscope. **Supplementary Figure 4.** Cellular viability affected by the treatment of examined compounds. **Supplementary Figure 5.** Subcellular localization of CMPK2. **Supplementary Figure 6.** Presentation of the full pictures of Fig. 6 A-3. **Supplementary Figure 7.** Knockout of CMPK2 did not affect mitochondrial mass. **Supplementary Figure 8.** Effects of CMPK2-KO on IFN-α-induced IL-1 production and caspase-1.

## Data Availability

The datasets used and/or examined for the current study will be available from the corresponding author on reasonable request.

## Abbreviations

SLE: Systemic lupus erythematosus
CMPK2: Cytidine/uridine monophosphate kinase 2
TDMs: THP-1-derived macrophages
oxLDL: Low-density lipoprotein
Dil-oxLDL: 1,19-Dioctadecyl-3,3,3′,3′-tetramethylindocarbocyanine perchlorate-oxLDL
DMEM: Dulbecco’s modified Eagle’s medium
FCS: Fetal calf serum
IFN-α: Interferon alpha
IFN-γ: Interferon gamma
Ab: Antibody
CCs: Cholesterol crystals
TLR: Toll-like receptor
LPS: Lipopolysaccharide
JAK: Janus kinase
Tyk2: Tyrosine kinase 2
MAPK: Mitogen-activated protein kinase
IL-1β: Interleukin-1β
SR-A: *Scavenger receptor* class-A
siRNA: Small interference RNA
PBMCs: Peripheral blood mononuclear cells
BMDMs: Bone marrow-derived macrophages
DCs: Dendritic cells
BODIPY: 4,4-Difluoro-1,3,5,7,8-pentamethyl-4-bora-3a,4a-diaza-s-indacene
ELISA: Enzyme-linked immunosorbent assay
PBS: Phosphate-buffered saline
PCR: Polymerase chain reaction
qPCR: Quantitative PCR
mtROS: Mitochondrial *reactive oxygen species*
*CCK-8*: Cell Counting Kit-8
WT: Wild type
