# Supplementary material for: Mitochondrial protein CMPK2 regulates IFN alpha-enhanced foam cell formation, potentially contributing to premature atherosclerosis in SLE
Source: Arthritis Res Ther. 2021 Apr 19;23:120. doi: 10.1186/s13075-021-02470-6 (PMC8054390; doi:10.1186/s13075-021-02470-6)
Supplement: Supplementary file 1 — Additional file 1: Supplementary Figure 1. Foam cell formation induced by different stimuli. Supplementary Figure 2. Induction of CMPK2 by different stimuli at 6 and 24 h time points. Supplementary Figure 3. Determination of CMPK2 localization with confocal microscope. Supplementary Figure 4. Cellular viability affected by the treatment of examined compounds. Supplementary Figure 5. Subcellular localization of CMPK2. Supplementary Figure 6. Presentation of the full pictures of Fig. 6 A-3. Supplementary Figure 7. Knockout of CMPK2 did not affect mitochondrial mass. Supplementary Figure 8. Effects of CMPK2-KO on IFN-α-induced IL-1 production and caspase-1. [file 13075_2021_2470_MOESM1_ESM.docx]

**Mitochondrial protein CMPK2 regulates IFN-alpha-enhanced foam cell formation, potentially contributing to premature atherosclerosis in SLE**

Jenn-Haung Lai ^a,b^, Li-Feng Hung ^c^, Chuan-Yueh Huang ^c^, De-Wei Wu ^a^, Chien-Hsiang Wu ^a^, Ling-Jun Ho ^c,*^

^a^ Department of Rheumatology, Allergy and Immunology, Chang Gung Memorial Hospital, Lin-Kou, Tao-Yuan, Taiwan, R.O.C.

^b^ Graduate Institute of Clinical Research, National Defense Medical Center, Taipei, Taiwan, R.O.C.

^c^ [Institute of Cellular and System Medicine](http://english.nhri.org.tw/inst_system/index.php), National Health Research Institute, Zhunan, Taiwan, R.O.C.

^*^ **Correspondence address**:

Ling-Jun Ho, PhD, [Institute of Cellular and System Medicine](http://english.nhri.org.tw/inst_system/index.php), National Health Research Institute, Zhunan, Taiwan, ROC. Tel.: +886-2-8791-8382; Fax: +886-2-8791-8382; E-mail: [lingjunho@nhri.org.tw](mailto:lingjunho@nhri.org.tw)

**Supplementary table and figure legends**

**
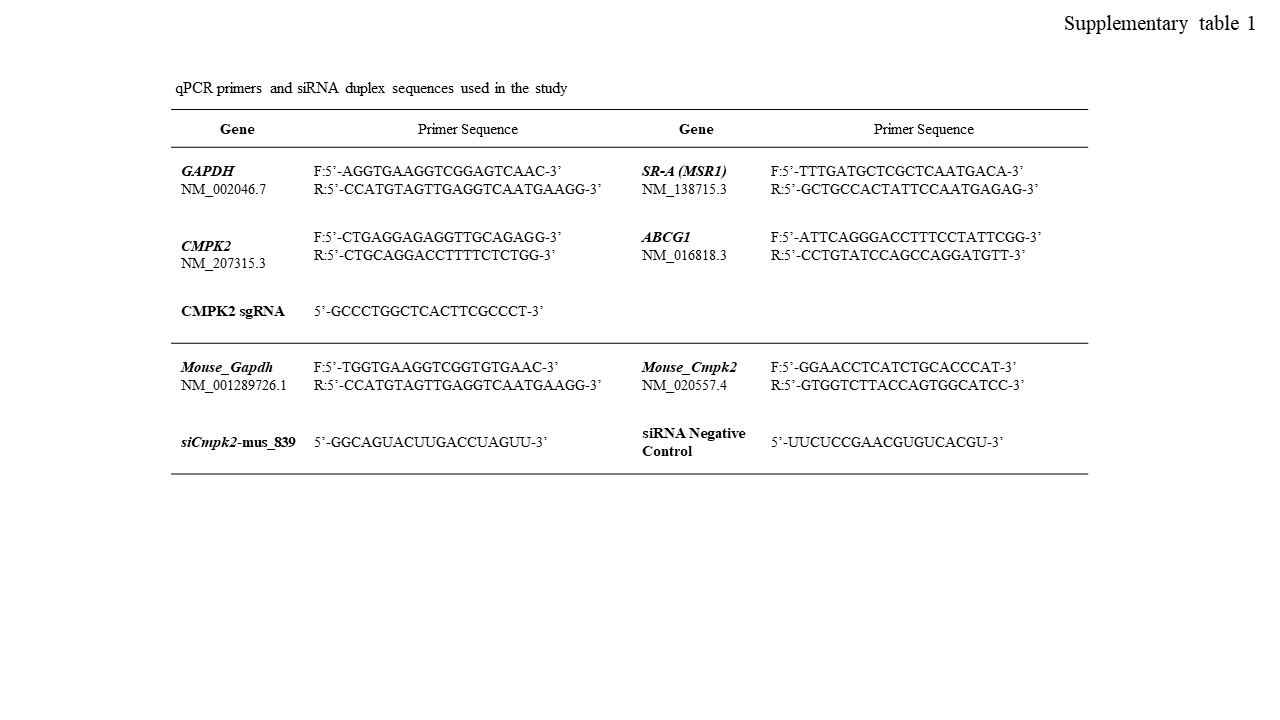
**

**
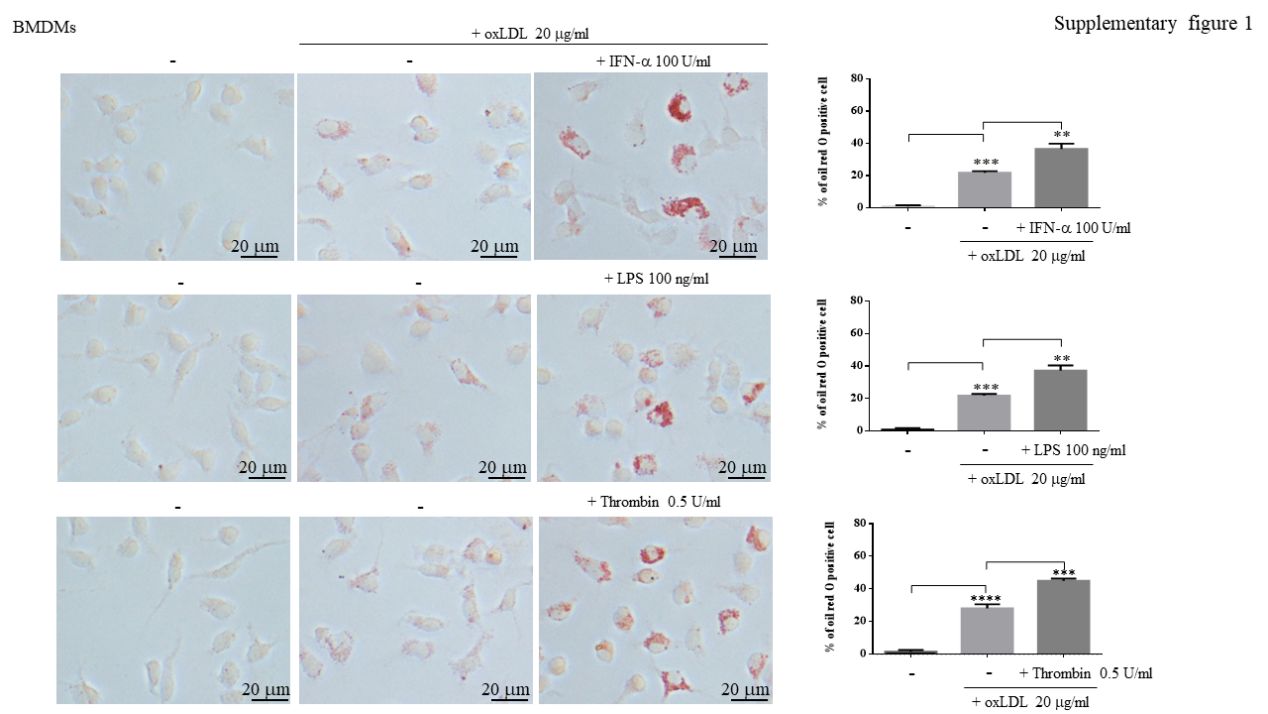
**

**Supplementary figure 1. Foam cell formation induced by different stimuli.** BMDMs were stimulated with oxLDL in the presence or absence of IFN-α, LPS or thrombin and foam cell formation was measured by oil red O staining (left). The cells were examined by light microscopy and the percentages of oil red O positive cells in 5 microscopic fields for each independent experiment were counted and calculated (right). Asterisks indicate values that are significantly different from the relevant control (*P < 0.05, **P < 0.01, ***P < 0.001 and ****P < 0.0001).

**
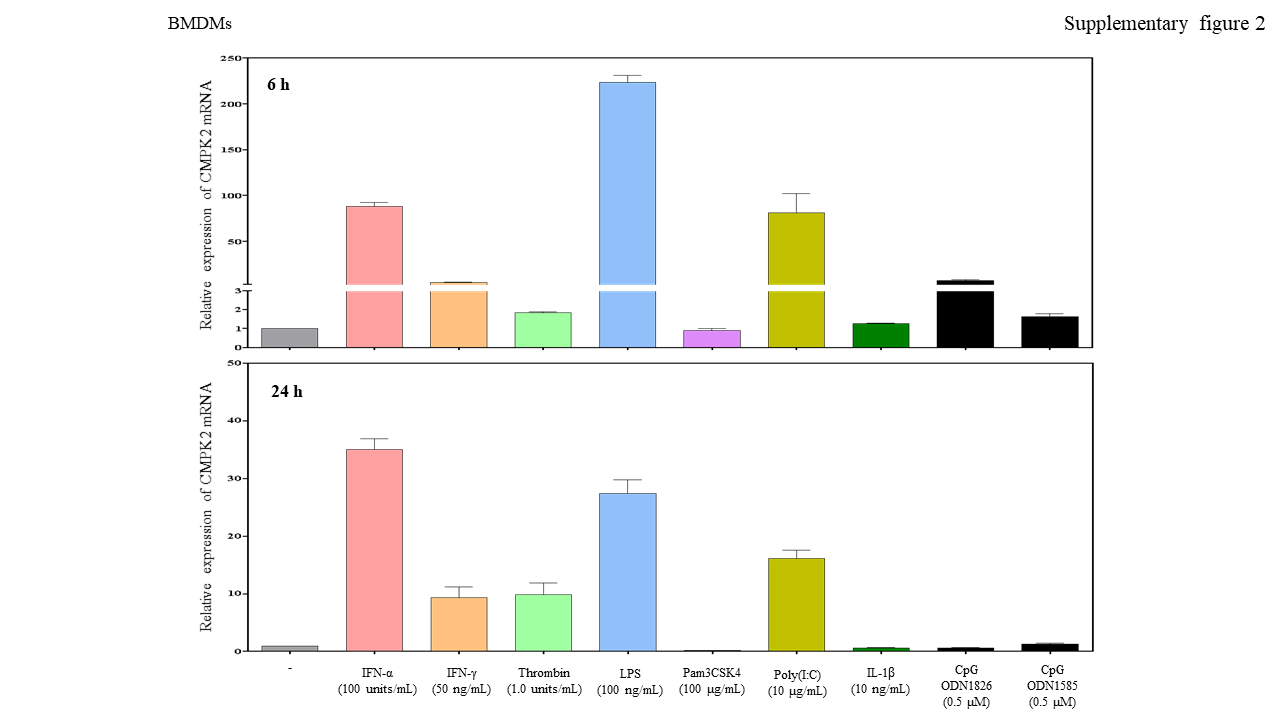
**

**Supplementary figure 2. Induction of CMPK2 by different stimuli at 6 and 24 h time points.** BMDMs were treated with different doses of IFN-α, IFN-γ, thrombin, LPS, Pam3CSK4, Poly(I:C), IL-1β, CpG ODN1826, or CpG ODN1585 for 6 h or 24 h, and the expression of CMPK2 mRNA was measured by qPCR. The representative results were shown.

**
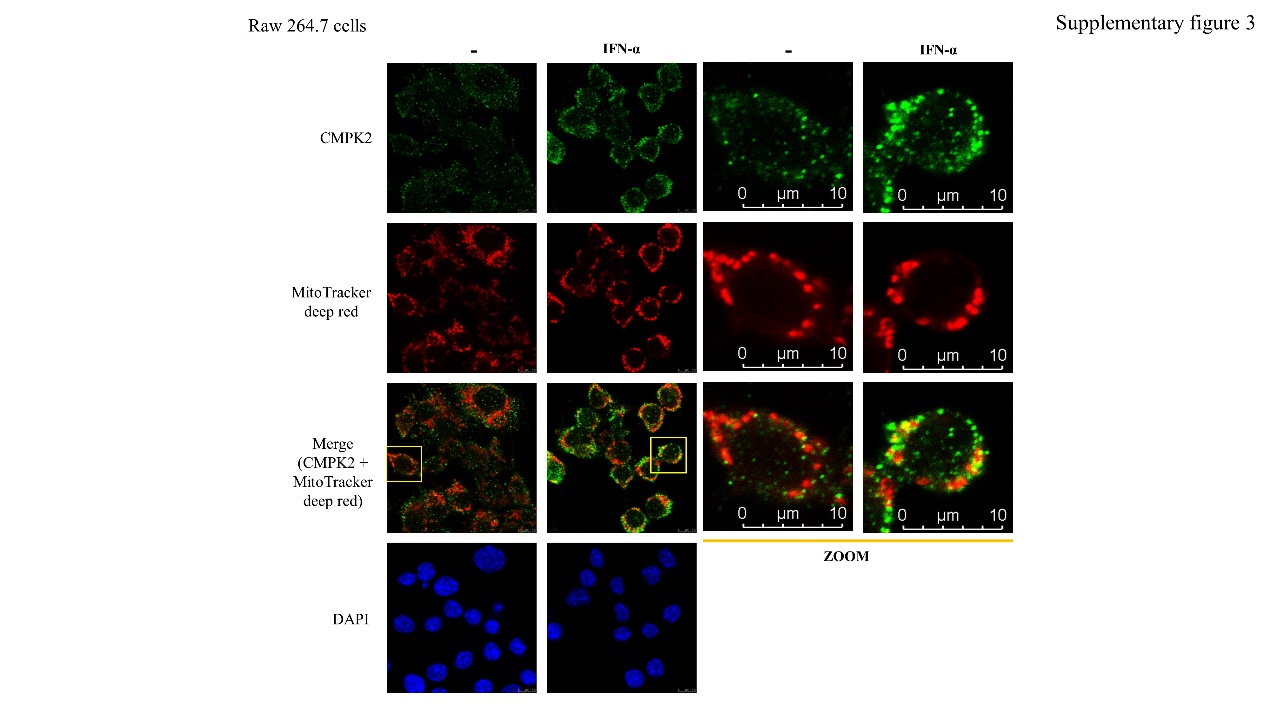
**

**Supplementary figure 3. Determination of CMPK2 localization with confocal microscope.** Raw264.7 cells were immunostained with anti-CMPK2, MitoTracker deep red and then counter stained with DAPI as described in Materials and Methods. The localization of CMPK2 and its relationship with MitoTracker deep red was determined. Representative pictures out of more than 3 independent experiments were shown.

**
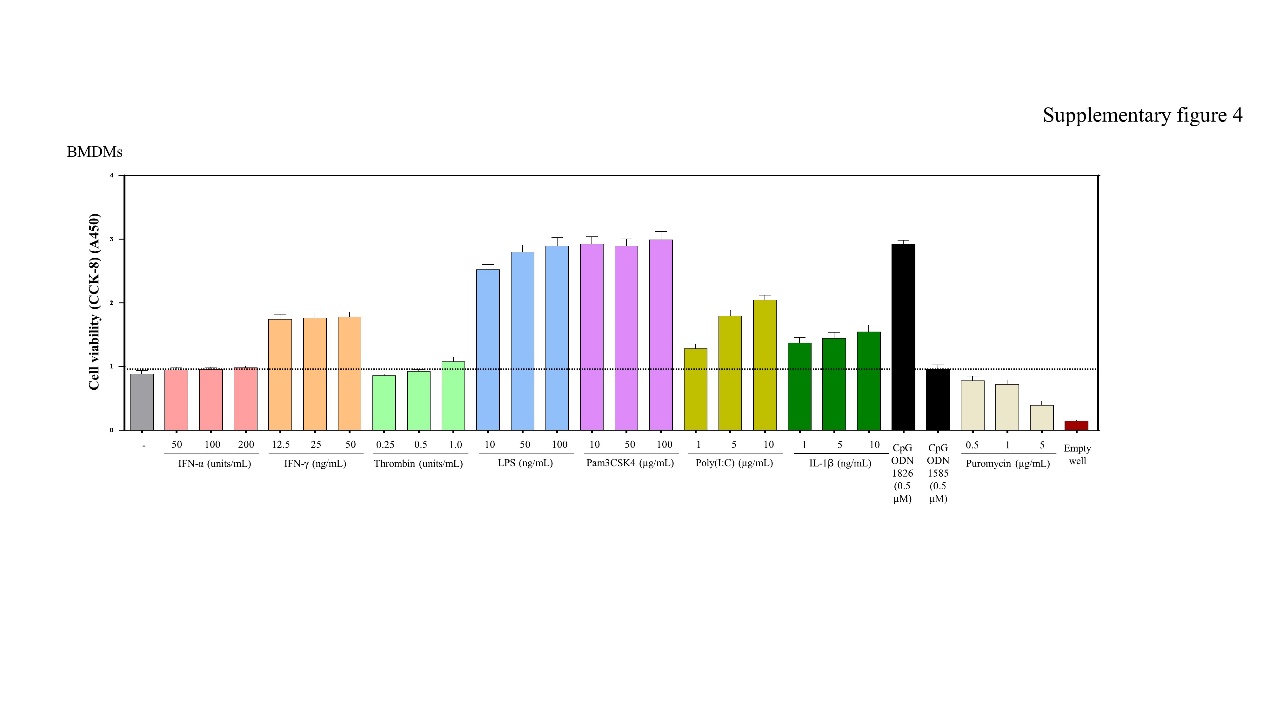
**

**Supplementary figure 4. Cellular viability affected by the treatment of examined compounds.** BMDMs were seeded in 96‐well plates and incubated overnight, and the medium was replaced with fresh culture medium containing the various stimulating agents indicated for 24 h. CCK‐8 reagent was added and incubated for 2 h at 37°C. The OD values for each well were read at a wavelength of 450 nm with a microplate reader. The relative cell viability in the stimulus-treated cells compared to that of the untreated cells is shown.

**
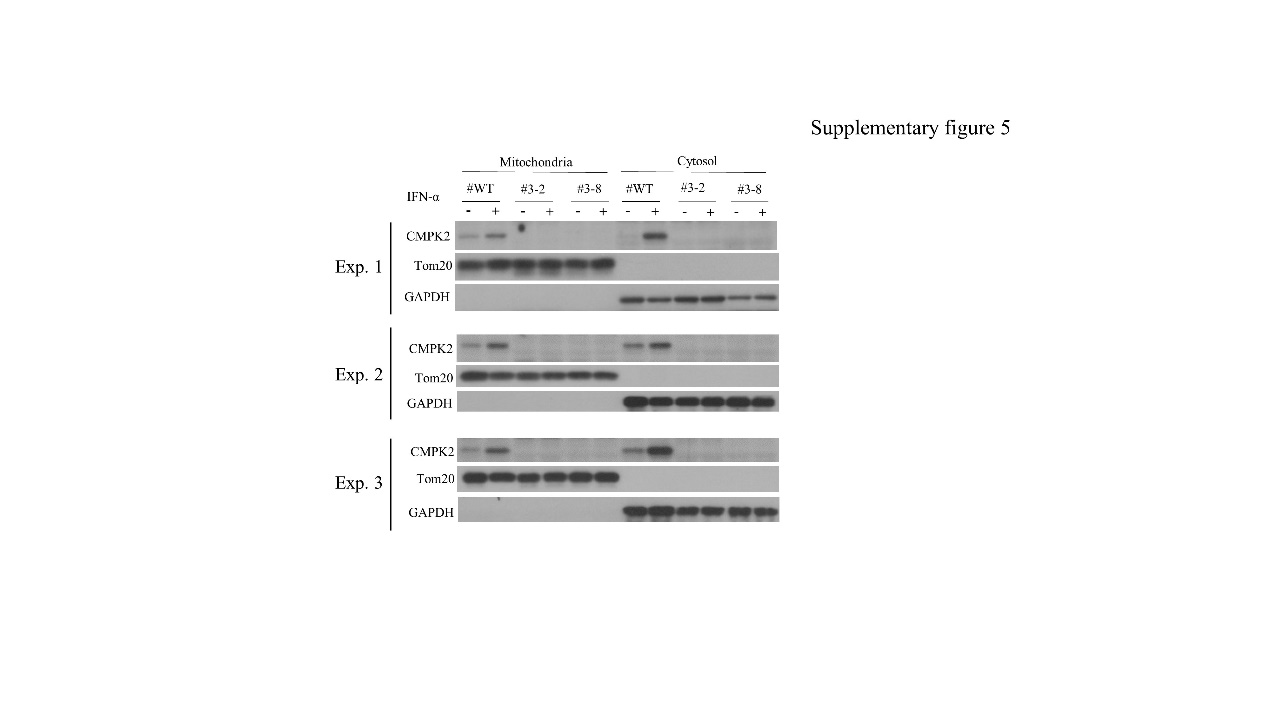
**

**Supplementary figure 5. Subcellular localization of CMPK2.** Wild-type TDMs (#WT) or CMPK2-KO clones #3-2 and #3-8 were treated with IFN-α or not for 24 h. Both mitochondrial and cytosolic fractions of cellular extracts were prepared as described in Materials and Methods. The levels of CMPK2, Tomm20 and GAPDH were measured by Western blotting accordingly. The results showed three independent experiments.

**
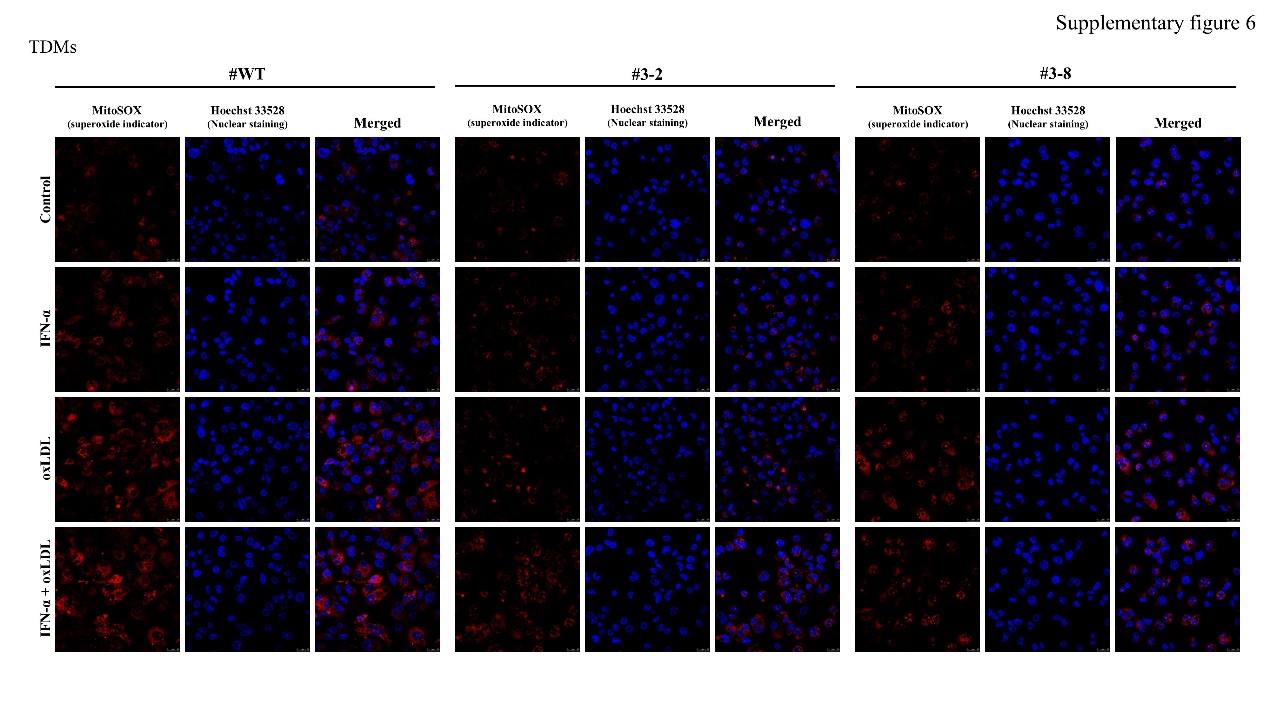
**

**Supplementary figure 6. Presentation of the full pictures of figure 6 A-3.** Wild-type TDMs (#WT) or CMPK2-KO clones #3-2 and #3-8 were treated with IFN-α, oxLDL or IFN-α+oxLDL for 24 h. The cells were then stained with MitoSOX and DAPI and examined under confocal microscopy.


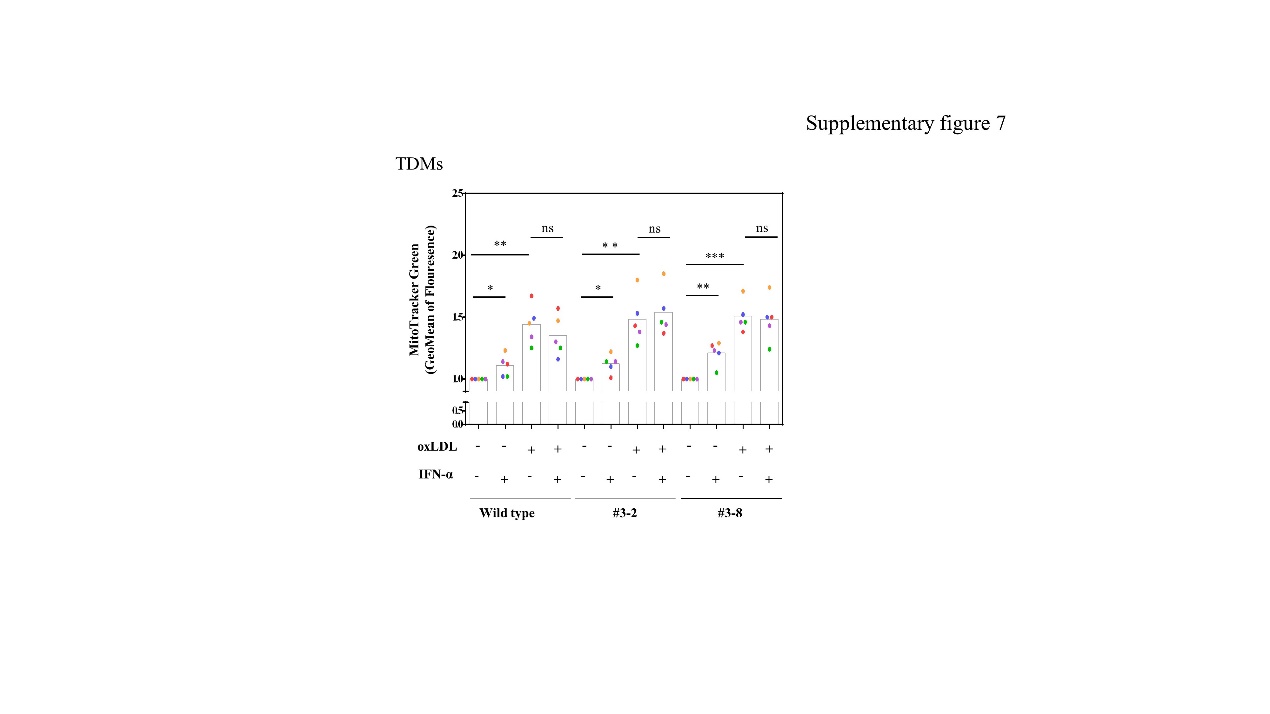


**Supplementary figure 7. Knockout of CMPK2 did not affect mitochondrial mass.** Wild-type TDMs (#WT) or CMPK2-KO clones #3-2 and #3-8 were treated with IFN-α, oxLDL or IFN-α+oxLDL for 24 h. Following the report in Cold Spring Harb Protoc. 2015 Sep 1;2015(9):830. doi: 10.1101/pdb.prot086298, the mitochondrial volume was measured with MitoTracker Green.

**
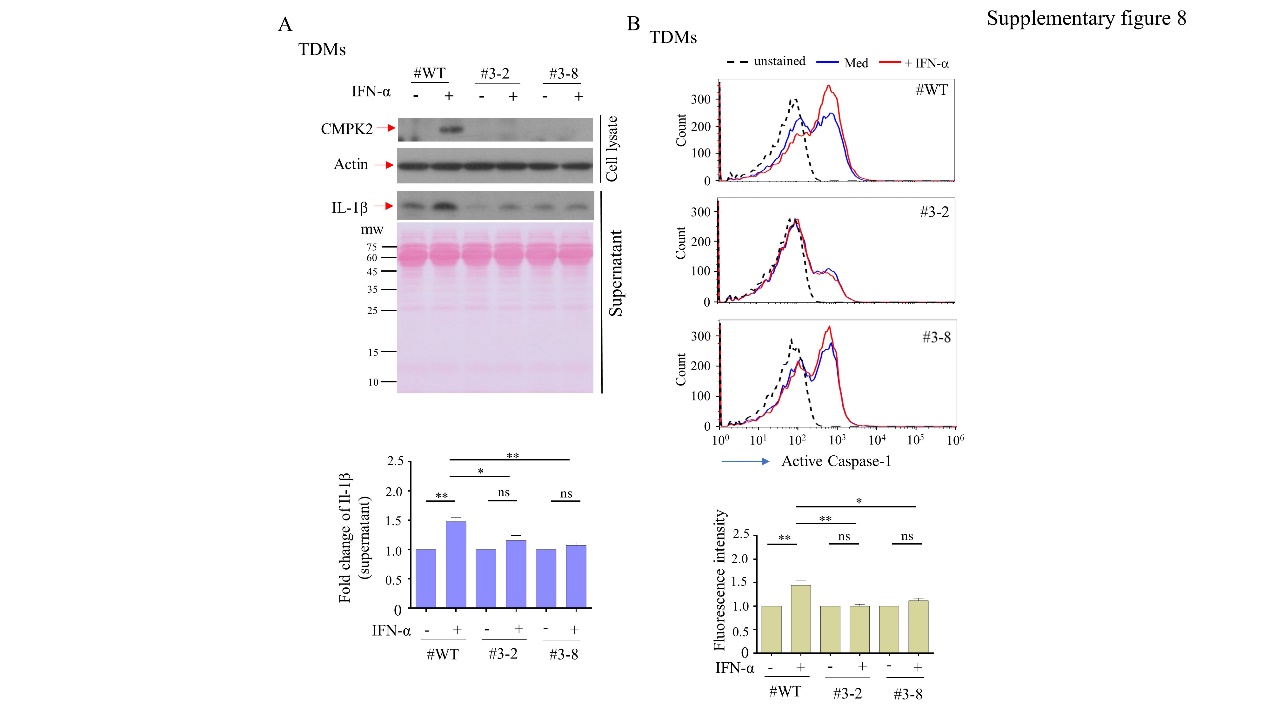
**

**Supplementary figure 8. Effects of CMPK2-KO on IFN-α-induced IL-1 production and caspase-1.** Wild-type cells and CMPK2-KO clones were stimulated with IFN-α, and the levels of IL-1β in the respective supernatant were measured by Western blotting and ponceau S staining (A, upper panel). The statistics from more than three independent experiments are shown (A, lower panel). The levels of active caspase-1 were determined by flow cytometry (B, upper panel), and the statistics from more than three independent experiments are shown (B, lower panel).
